# PCA22 acts as a suppressor of *atrzf1* to mediate proline accumulation in response to abiotic stress in Arabidopsis

**DOI:** 10.1093/jxb/erx069

**Published:** 2017-03-21

**Authors:** Ah-Reum Kim, Ji-Hee Min, Kyeong-Hwan Lee, Cheol Soo Kim

**Affiliations:** 1 Department of Plant Biotechnology, Chonnam National University, Gwangju 61186, Republic of Korea; 2 Department of Rural and Biosystems Engineering, Agricultural Robotics and Automation Research Center, Chonnam National University, Gwangju 61186, Republic of Korea

**Keywords:** Abiotic stress response, AtRZF1, *pca* mutant, pollen tube length, proline metabolism, suppressor.

## Abstract

Proline metabolism is important for environmental responses, plant growth, and development. However, its precise roles in plant abiotic stress tolerance are not well understood. Mutants are valuable for the identification of new genes and for elucidating their roles in physiological mechanisms. We applied a suppressor mutation approach to identify novel genes involved in the regulation of proline metabolism in Arabidopsis. Using the *atrzf1* (*Arabidopsis thaliana ring zinc finger 1*) mutant as a parental line for activation tagging mutagenesis, we selected several mutants with suppressed induction of proline accumulation under dehydration conditions. One of the selected mutants [*proline content alterative 22* (*pca22*)] appeared to have reduced proline contents compared with the *atrzf1* mutant under drought stress. Generally, *pca22* mutant plants displayed suppressed *atrzf1* insensitivity to dehydration and abscisic acid during early seedling growth. Additionally, the *pca22* mutant exhibited shorter pollen tube length than wild-type (WT) and *atrzf1* plants. Furthermore, *PCA22*-overexpressing plants were more sensitive to dehydration stress than the WT and RNAi lines. Green fluorescent protein-tagged PCA22 was localized to the cytoplasm of transgenic Arabidopsis cells. Collectively, these results suggest that *pca22* acts as dominant suppressor mutant of *atrzf1* in the abiotic stress response.

## Highlight

PCA22 negatively regulates the drought response through the modulation of osmolytic components and regulates pollen tube length in Arabidopsis.

## Introduction

Proline (Pro) is a multifunctional amino acid in plants, and Pro accumulation has been reported to occur in response to drought, high salinity, heavy metals, pathogen infection, low temperature, and oxidative stress (Verbruggen and Hermans, 2008; Yang *et al.*, 2009; Szabados and Savouré, 2010). Pro accumulation is believed to play adaptive roles in plant stress tolerance. Pro has been proposed to act as a compatible solute and a source of nitrogen and carbon storage (Hare and Cress, 1997). Pro has also been proposed to function as a molecular chaperone to stabilize the structure of proteins, and also as a regulator of cellular redox potential, and an antioxidant controlling free radical levels (Hare *et al.*, 1999). Recently Ju *et al.* (2013) reported that Pro accumulation is regulated by ubiquitination during abiotic stress. Finally, Pro accumulation may be part of the stress signal influencing the adaptive response (Maggio *et al.*, 2002).

Protein ubiquitination is an important post-translational modification, which is employed by eukaryotes to regulate diverse cellular and developmental processes (Dye and Schulman, 2007). Although understanding of ubiquitination in regards to plant responses to abiotic stresses has been supplemented with new reports in recent years, many gaps in knowledge on this process still exist. Among the genes whose roles have not been explained to date is the *Arabidopsis thaliana RING Zinc Finger 1* (*AtRZF1*) gene. It encodes a RING (for Really Interesting New Gene)-type subunit of the E3 ubiquitin ligase family. Recently, a number of Arabidopsis RING E3 ligases were shown to be involved in various cellular processes, such as auxin signaling, abscisic acid (ABA) signaling, brassinosteroid response, seed germination, seedling development, adaptive pathway to nitrogen limitation, and sugar responses (Stone *et al.*, 2006; Peng *et al.*, 2007; Bu *et al.*, 2009; Santner and Estelle, 2009; Huang *et al.*, 2010). In particular, RING proteins play a key role in the response to environmental stimuli. For example, they participate in photomorphogenesis, defense signaling, and senescence, and in tolerance mechanisms against cold, drought, salt, and osmotic stress (Yan *et al.*, 2003; Craig *et al.*, 2009; Fujita *et al.*, 2011; Smirnova *et al.*, 2011).

The product of the *AtRZF1* gene plays an important role in the drought response (Ju *et al.*, 2013). Water deficit response assays have indicated that, while the *atrzf1* mutant is less sensitive to drought, *AtRZF1*-overexpressing plants are more sensitive, suggesting that AtRZF1 negatively regulates the drought response during seed germination and early seedling development. The accumulation of Pro in *atrzf1* plants was greater than in wild-type (WT) and *AtRZF1*-overexpressing plants, which might suggest that AtRZF1 is responsible for the induction of leaf drought sensitivity through the modulation of osmolytic components (Ju *et al.*, 2013). Considering that RING-type E3 ubiquitin ligase is involved in many aspects of the drought signaling network, there is still a need to explain the exact role of AtRZF1 and to identify new components that interact with AtRZF1 in the signaling pathway.

To obtain insight into the mechanism of AtRZF1 action in the regulation of Pro metabolism and abiotic stress signal transduction in young seedlings, a genetic screen was performed to identify suppressors of *atrzf1* under drought conditions. Suppressor mutants were generated after T-DNA activation tagging mutagenesis, and were selected based on decreased accumulation of Pro in response to drought stress, which increased levels in its parental line, the *atrzf1* mutant. Here, we present detailed characteristics of one suppressor mutant of *atrzf1*, termed *pca22* (*proline content alterative 22*), in the presence of mannitol, polyethylene glycol (PEG), and ABA during early seedling growth, as a first step in the identification of the suppressor gene.

## Materials and methods

### Plant materials, growth conditions, and stress induction

*Arabidopsis thaliana* Columbia (Col-0) and *atrzf1* were used as the WT and background lines for suppressor screening, respectively. To observe phenotypic changes, the seeds were sown directly in pots containing a vermiculite:soil (1:2) mixture under growth room conditions (16 h light/8 h dark, 21 ± 2 °C, and 60 ± 5% relative humidity). Otherwise, the seeds were sown onto Murashige and Skoog (MS) plates containing 0.8% agar after sterilization with 75% ethanol containing 0.05% Tween-20 for 10 min. Ten-day-old Arabidopsis seedlings were challenged with dehydration stress by submersion in a solution containing 10% PEG [electrical conductivity (EC)=63.8 μS cm^−1^ and ψs= −0.34 MPa] or 400 mM mannitol (EC=1.2 μS cm^−1^ and ψs= −1.2 MPa). Samples were collected following 0, 3, and 6 h dehydration stress. For ABA, 10-day-old seedlings were submerged in a solution containing 100 μM ABA as previously described (Zeller *et al.*, 2009) and sampled at 0, 3, and 6 h. For drought stress, seedlings were grown in pots subjected to normal watering every 4 d. After 10 d, the plants were divided into two groups for stress treatments. One group was subjected to drought stress (relative water content=69.34 ± 3.6%) by withholding water for 12 d, and a control group was watered normally. In each case, the retrieved seedlings were immediately frozen in liquid nitrogen and stored at −80 °C.

### *Generation of activation-tagged* atrzf1 *transgenic plants and selection of* pca *mutants*

*atrzf1* mutants were transformed with *Agrobacterium tumefaciens* strain GV3101 containing the pSKI015 plasmid (Weigel *et al.*, 2000) by the vacuum infiltration method (Bechtold and Pelletier, 1998). Seeds collected from infiltrated *atrzf1* mutants were sterilized with sodium hypochlorite, stored at 4 °C for 2 d, and sown directly in soil. Activation-tagged transgenic *atrzf1* mutants (T_1_ generation plants) were selected by spraying 0.5% BASTA (Duchefa, Haarlem, The Netherlands) once every 3 d for 2 weeks, and were visually inspected to identify plants exhibiting WT-like morphology. We selected ~26 000 BASTA-resistant T_1_ generation lines. Because *atrzf1* is known for the hyperaccumulation of Pro in response to water deficit conditions, the T_1_ population was screened for decreased Pro concentration to drought stress compared with the *atrzf1* mutant. To isolate *pca* mutants, the pattern of Pro accumulation was initially assessed in excised leaves from 5-week-old T_1_ plants and *atrzf1* mutants subjected to drought-induced Pro accumulation following 7 d drought. Nine putative suppressor mutants were identified in the first screen and were grown to produce seeds. BASTA resistance in T_2_ generation plants from selected suppressor mutants segregated as a single locus. Then, seeds of putative suppressors (T_4_) were sown on dehydration medium (400 mM mannitol and 10% PEG) to confirm the sensitive phenotype. After analyses, nine homozygous lines with suppressed *atrzf1* insensitivity to dehydration conditions during cotyledon greening were isolated; one of these (*pca22*) is characterized in the present study.

### Determination of Pro and malondialdehyde (MDA) contents

Pro contents were measured as previously described (Bates *et al.*, 1973). Briefly, Pro was extracted from 100 mg of plant leaves by grinding in 1 ml of 3% sulfosalicylic acid. A 200 μl aliquot of extract was reacted with 100 μl of the ninhydrin reagent mixture (80% glacial acetic acid, 6.8% phosphoric acid, and 70.17 mM ninhydrin) for 60 min at 100 °C. An ice bath was used to terminate the reaction. The reaction mixture was extracted with 200 μl of toluene and vortexed. Absorbance of the toluene layer was read at 520 nm in a UV/VIS spectrophotometer (JASCO, Tokyo, Japan). The Pro concentration was extrapolated from a standard curve, and calculated on an FW basis as follows: [(ng proline ml^–1^×ml extraction buffer)/115.5 ng nmol] g^–1^ sample=nmol proline g^–1^ FW material.

The level of membrane damage was determined by measuring MDA as an end-product of membrane lipid peroxidation as previously described (Hodges *et al.*, 1999; Zhang *et al.*, 2012). The samples were homogenized in 10% (w/v) trichloroacetic acid (TCA) solution on ice. The homogenate was centrifuged for 5 min at 10 000 rpm and the supernatant was collected. A 1 ml aliquot of 10% (w/v) TCA containing 0.6% (w/v) thiobarbituric acid (TBA) was added to a 0.5 ml aliquot of the supernatant. The mixture was then maintained for 15 min in a boiling water bath and then quickly cooled on ice. The absorbance of the colored supernatant was measured at 532 nm (*A*_532_) and was corrected for non-specific absorbance at 450 nm (*A*_450_) and 600 nm (*A*_600_). The MDA content was calculated with the equation: MDA=6.45(*A*_532_−*A*_600_)−0.56×*A*_450_ (Hodges *et al.*, 1999; Zhang *et al.*, 2012).

### Measurement of relative water content

For relative water content values, detached rosette leaves were placed on open-lid Petri dishes at room temperature with 60% humidity under dim light. The weights of rosette leaves were measured at various times. Leaf water content was expressed as the percentage of initial fresh weight.

### Phenotype analysis and stress tests

For each comparison, seeds of all genotypes were planted in the same plate containing MS medium with or without mannitol (400 mM), PEG (10%), or ABA (1 μM). For each experiment, three technical replicates were performed. Seeds used for these experiments were harvested and stored. Cotyledon greening was recorded on days 9–12 after the seeds were sown, depending on the experiment. Cotyledon greening was defined as visible expansion and greening of the cotyledon. Each experiment included 50 seeds of each genotype.

### Extraction of RNA, quantitative real-time PCR (qPCR), and reverse transcription–PCR (RT–PCR)

Total RNA was extracted from the frozen samples using the Plant RNeasy extraction kit (Qiagen, Valencia, CA, USA). To remove any residual genomic DNA in the preparation, the RNA was treated with RNase-free DNase I in accordance with the manufacturer’s instructions (Qiagen). The concentration of RNA was quantified accurately via spectrophotometric measurements, and 5 μg of total RNA was separated on 1.2% formaldehyde agarose gels to determine the concentration and monitor the integrity.

qPCR was carried out with a Rotor-Gene 6000 quantitative PCR apparatus (Corbett Research, Mortlake, NSW, Australia) and the results were analyzed using RG6000 1.7 software (Corbett Research). Total RNA was extracted from treated 10-day-old Arabidopsis seedlings using an RNeasy Plant Mini kit (Qiagen). qPCR was carried out using the SensiMix One-Step kit (Quantance, London, UK). Arabidopsis *Actin1* (*ACT1*) was used as the internal control. Quantitative analysis was carried out using the ΔΔ-C_T_ method (Livak and Schmittgen, 2001). Each sample was run in three independent experiments. The reaction primers utilized are shown in Supplementary Table S1 at *JXB* online.

RT–PCR was employed to measure the levels of *At2g28620*, *At2g28625* (*PCA22*), *At2g28630* [*Ketoacyl-CoA Synthase 12* (*KCS12*)], *AtRZF1*, and *ACT1* expression in plants of each genotype. A 500 ng aliquot of total RNA was used in the RT–PCR, and the reaction primers utilized are shown in Supplementary Table S1. After 27 PCR amplification cycles, 20 µl of each RT–PCR product were loaded onto a 1.2% (w/v) agarose gel in order to visualize the amplified DNAs.

### Determining the location of the T-DNA insertion in genomic DNA by TAIL-PCR

Genomic DNA was isolated from *pca22* mutants and thermal asymmetric interlaced (TAIL)-PCR was performed using arbitrary degenerate (AD) and T-DNA left border (LB) end primers (Liu *et al.*, 1995) (Supplementary Table S1). Purified fragments following tertiary TAIL-PCR were cloned into the pGEM T-easy vector (Promega, Madison, WI, USA) for DNA sequence analysis, and the flanking sequences obtained were used to perform a BLAST search using the NCBI program (http://www.ncbi.nlm.nih.gov). Finally, specific primers were designed (Supplementary Table S1) and used in combination with the T-DNA-LB primer to amplify specific fragments, which were subsequently sequenced to confirm the T-DNA insertion site.

### *Generation of* PCA22 *transgenic lines*

Total RNA was isolated from Arabidopsis leaves using TRIzol reagent (Invitrogen, Carlsbad, CA, USA). To construct the *PCA22* overexpression lines, the full-length *PCA22* cDNA (*At2g28625*) was amplified using RT–PCR and the generated product was cloned into the pDONR/ZEO vector (Invitrogen) for DNA sequence analysis. The RT–PCR primers were as follows: forward 5'- GGGGACAAGTTT GTACAAAAAAGC AGGCTTCATGAACG AAGAGGAGAGAGTTGG-3' and reverse 5'- GGGGACCAC TTTGTACAAGA AAGCTGGGT CACAAAGGCAA CCACAACATCTCT-3'. Amplification proceeded for 35 cycles consisting of 94 °C for 30 s; 57 °C for 30 s; and 72 °C for 1 min. The PCR-amplified products were then directionally cloned into the plant expression vector pGWB514. The resultant construct was introduced into *Agrobacterium tumefaciens* strain GV3101 and the *atrzf1* mutant for the complementation lines, and into Arabidopsis (Col-0) for overexpression lines, via *in planta* vacuum infiltration. T_3_ homozygous transgenic lines were selected for phenotypic characterization. Hygromycin B (A.G. Scientific, San Diego, CA, USA) resistance of the T_2_ generation from these selected lines segregated as a single locus.

To generate the *PCA22* RNAi lines, the gene-specific cDNA fragment of *PCA22* was amplified by PCR using the forward primer 5'-GGGGACAAGTTTGTACAAAAAAGCAGGCTT CATGAACGAAGAGGAGAGAGTTGG-3' and reverse primer 5'-GGGGACCACTTTGTACAAGAAAGCTGGG TCACAAAGGCAACCACAACATCTCT-3'. The PCR products were initially cloned into the entry vector pDONR/ZEO and confirmed by sequencing. Then, this RNAi *PCA22* cDNA construct was subcloned into the destination vector pB7GWIWG2ii (Karimi *et al.*, 2002) under the control of the constitutive 35S promoter. The construct was then transformed into Arabidopsis plants, and the resultant T_3_ homozygous transgenic *PCA22* RNAi lines (*ri14* and *ri27*) were evaluated for ABA and abiotic stress sensitivity.

### Localization of PCA22–green fluorescent protein (GFP) fusion proteins in transgenic plants

To analyze the subcellular localization of the PCA22 protein in transgenic Arabidopsis, the *PCA22* cDNA fragment was amplified using the following primers: 5'-GGGGACAAGTTTGTACAAAAAA GCAGGCTTCATGAACGAAGAGGAGAGAGTTGG-3' and 5'-GGGGACCACTTTGTACAAGAAAGCTGGGTCAC AAAGGCAACCACAACATCTCT-3' on the basis of the sequence information deposited in a cDNA database (NCBI, http://www.ncbi.nlm.nih.gov). PCR products were inserted into the pEarleyGate103 vector under the control of the constitutive 35S promoter. The nucleotide sequence of the new construct was confirmed by DNA sequencing. To determine the intracellular localization of PCA22–GFP fusion proteins in transgenic plants, root samples were mounted on microscope slides and observed using a FluoView1000 confocal microscope (Olympus, Tokyo, Japan). Confocal images were obtained and processed using FV10-ASW 1.7A computer software (Olympus).

### Measurement of pollen tube elongation

Pollen germination *in vitro* followed a modified version of the protocol described by Li *et al.* (1999). In brief, pollen harvested from newly fully opened flowers was placed on the surface of pollen germination medium [1 mM Ca(NO_3_)_2_, 1 mM CaCl_2_, 1 mM MgSO_4_, 1.5 mM boric acid, 0.5% (w/v) agarose, 18% (w/v) sucrose, pH 7] and incubated at 28 °C for 12 h. Pollen grains obtained from WT, *atrzf1* mutant, *pca22* mutant, and *PCA22* transgenic plants were germinated for 12 h *in vitro*. Pollen tube length was determined from digital micrographs acquired via light microscopy. Three independent replicates were performed, and for each replicate at least 150 pollen tubes were assessed to obtain a mean value for subsequent statistical analyses.

### Statistical analysis

Statistical analyses were performed using Excel and SPSS (Ver. 21.0, IBM, USA). ANOVA was used to compare statistical differences based on the Student’s *t*-test, at a significant level of 0.01<*P*<0.05, or *P*<0.01.

## Results

### *The* pca22 *mutant obtained from activation tagging*

**Fig. 1.**
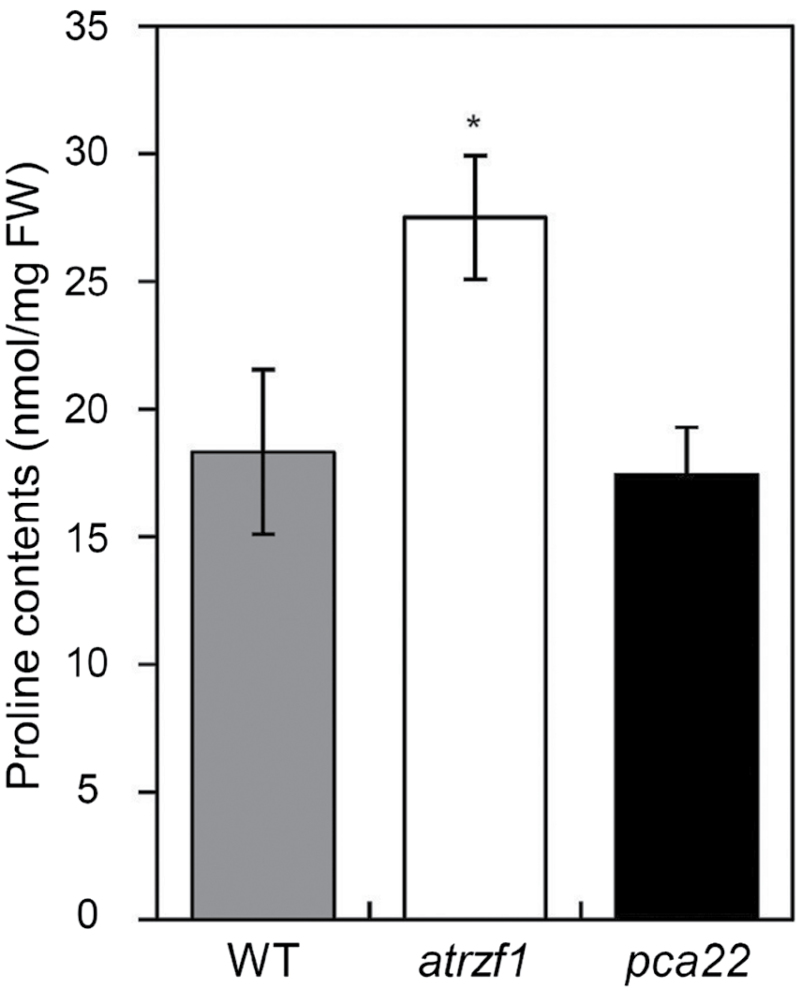
Proline content in the leaves of WT, *atrzf1*, and *pca22* plants. Light-grown 5-week-old plants were grown for 7 d without watering. Leaf tissues were carefully excised after drought treatment, and their proline content was measured. Error bars represent the SD. Differences among WT, *atrzf1*, and *pca22* plants grown under the same conditions are significant at 0.01<*P*<0.05 (*).

In order to determine the key genes involved in cellular Pro metabolism in Arabidopsis, *pca* mutants were generated and characterized under dehydration conditions. Because *atrzf1* is known to hyperaccumulate Pro in response to water deficit conditions (Ju *et al.*, 2013), the activation-tagged T_1_ population was screened for decreased Pro concentration to drought stress compared with the *atrzf1* mutant. *atrzf1* mutants were transformed with *Agrobacterium* harboring the binary vector pSKI015, which contained four copies of the *Cauliflower mosaic virus* (CaMV) 35S enhancer at the right border of the T-DNA and a *BAR* (*Bialaphos resistance*) gene as a selectable marker (Weigel *et al.*, 2000). Transformants were selected on soil sprayed with BASTA and subsequently screened under drought condition (see the Materials and methods). Approximately 26 000 activation-tagged *atrzf1* transgenic lines were screened and nine mutants were selected during drought stress. One of the mutant lines, *pca22*, was analyzed for further physiological and molecular characterization. Pro accumulation in the *pca22* mutant was found to be similar under water deficit conditions to that of the WT, while the Pro content of *pca22* was significantly lower than that of the *atrzf1* mutant (Fig. 1). These results suggest that *pca22* participates in the suppression of Pro production in *atrzf1* mutants under drought conditions.

### *Abiotic stress responses of the* pca22 *mutant*

**Fig. 2.**
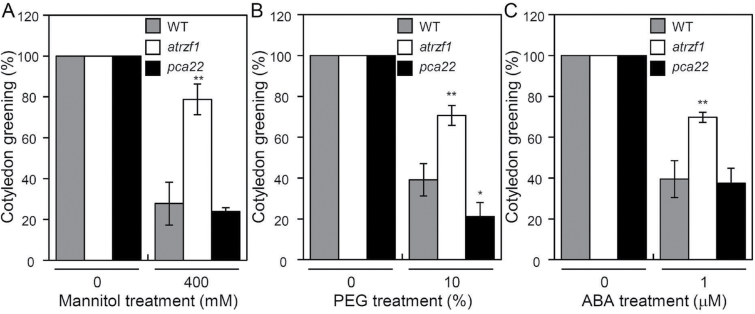
Effect of the *pca22* mutant on sensitivity to dehydration stress and ABA sensitivity. Effect of osmotic stress and ABA on cotyledon greening. Seeds were sown on MS agar plates supplemented with mannitol, PEG, or ABA and permitted to grow for 8 d; seedlings with green cotyledons were then counted (triplicates, *n*=50 each). Error bars represent the SD. Differences among WT, *atrzf1*, and *pca22* plants grown under the same conditions are significant at 0.01<*P*<0.05 (*) or *P*<0.01 (**).

Ju *et al.* (2013) demonstrated that AtRZF1, an E3 ubiquitin ligase, is involved in the drought response. Because the *pca22* mutant was obtained from activation-tagged *atrzf1* transgenic lines, it is likely to be related to abiotic stress responses. To determine whether *pca22* could be associated with abiotic stress responses, we examined cotyledon greening in WT, *atrzf1*, and *pca22* seedlings in the presence of mannitol, PEG, or ABA. The germination rate among the WT, *atrzf1*, and *pca22* was similar and not poor in MS medium (Fig. 2; Supplementary Fig. S3B). In addition, developmental processes were not affected in the *pca22* mutant (data not shown). Comparison of the *pca22* and *atrzf1* mutants demonstrated that significantly fewer cotyledons expanded and turned green after 8 d germination when grown on MS medium containing 400 mM mannitol, 10% PEG, or 1 μM ABA, whereas the cotyledon greening rate was similar between WT and *pca22* seedlings, except under the 10% PEG condition (Fig. 2). These results showed that the *pca22* mutant was more likely to be sensitive to abiotic stresses than the *atrzf1* mutant. To evaluate further the response to drought stress, cut rosette water loss rates of the plants were estimated. To assess water loss from leaves, leaves of similar size, age, and positions on the WT, *atrzf1*, and *pca22* were detached and measured for decreases in fresh weight, as described previously (Sang *et al.*, 2001). After detachment, leaves from the *pca22* mutant exhibited higher loss of fresh weight than those from *atrzf1* through an experimental time course, while the rate of water loss was similar in WT and *pca22* plants (Supplementary Fig. S1).

In order to investigate the molecular changes associated with dehydration stress, we evaluated four genes, *Responsive to Desiccation 29B* (*RD29B*), *Responsive to ABA 18* (*RAB18*), *Delta 1-pyrroline-5-carboxylate reductase* (*P5CR*), and *Delta 1-pyrroline-5-carboxylate synthase* (*P5CS*), which are induced by various abiotic stresses (Sharma and Verslues, 2010; Msanne *et al.*, 2011). The transcript levels of dehydration-inducible genes were more markedly reduced in *pca22* and WT lines than in *atrzf1* mutants following mannitol treatment; however, the expression of the four genes was similar in WT and *pca22* plants (Supplementary Fig. S2A–D). These observations support the notion that PCA22 regulates the expression of AtRZF1-mediated stress marker genes under dehydration stress conditions. Satoh *et al.* (2004) demonstrated that AtbZIP11, a basic leucine-zipper transcription factor, is involved in hypo-osmolarity- and Pro-responsive expression of *Pro dehydrogenase* (*PDH*). The AtMYB2 transcription factor participates in the up-regulation of *P5CS1* transcription (Yoo *et al.*, 2005). As shown in Supplemetnary Fig. S2E and F, rehydration-induced expression of *AtbZIP11* was significantly reduced in the *atrzf1* mutant, in comparison with that in WT and *pca22* plants under dehydration conditions; however, the transcript level of *AtMYB2* increased more in *atrzf1* than in WT and *pca22* plants. These results indicate that molecular events associated with Pro content were reduced more in *pca22* than in the *atrzf1* mutant.

### *Location of the T-DNA insertion in the* pca22 *mutant*

**Fig. 3.**
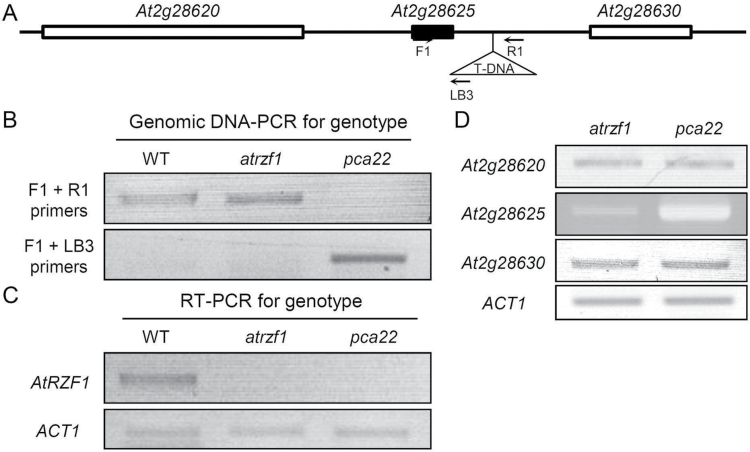
Identification of 35S enhancer elements containing the T-DNA insertion region in *pca22*. (A) A map of the T-DNA insertion of *pca22* on chromosome 2. The structure of the activation-tagged locus of *pca22* is shown in the flanking region containing *At2g28625* and *At2g28630*. The arrows mark the positions of gene-specific primers and a T-DNA LB-specific primer used for PCR amplification. (B) T-DNA linkage analysis of the *pca22* phenotype. Genomic PCR using the gene-specific primers F1 and R1 yielded a DNA band specific for the WT and *atrzf1* lines at the *PCA22* locus, whereas PCR using primers F1 and LB3 produced a *pca22*-specific band. (C) RT–PCR to compare the transcript levels of *AtRZF1* and *ACT1* control genes in WT, *atrzf1*, and *pca22* mutants. (D) Expression of the three neighboring genes (*At2g28620*, *At2g28625*, and *At2g28630*) encoding a functional protein near the activation-tagged locus was analyzed in *pca22* plants and compared with those in *atrzf1* by RT–PCR. *ACT1* was used as an internal control for RT–PCR.

To identify the position of the T-DNA insertion site and the activated gene, TAIL-PCR was performed on the *pca22* mutant using primers from both ends of the LB and AD primers (Supplementary Table S1). The sequences flanking the LB region obtained from analysis of the TAIL-PCR products were subjected to a BLAST search, and a single insertion site was mapped in *pca22*. The T-DNA insertion in *pca22* was located on the intergenic region between the two genes, *At2g28625* and *At2g28630*, which encode an unknown protein and KCS12, respectively (Fig. 3A). The T-DNA locus in *pca22* was found close to *At2g28625* (Fig. 3A). To characterize further the T-DNA-associated DNA sequence, specific primers (F1 and R1) were designed (Supplementary Table S1) and used in combination with T-DNA-specific LB3 primers to confirm the insertion site within the WT, *atrzf1*, and *pca22* genome (Fig. 3A, B). To genotype the locus, PCR primers F1 and R1 were used to amplify the WT and *atrzf1* sequences, while primers F1 and LB3 were used to detect the *pca22* mutant. Using the F1–LB3 primer pair, only the *pca22* mutant but not the WT or *atrzf1* mutant could be amplified by PCR (Fig. 3B). Furthermore, RT–PCR indicated that the *AtRZF1* transcript accumulated in 10-day-old WT seedlings, but was not detectable in *atrzf1* and *pca22* mutant seedlings (Fig. 3C). PCR-based genotyping analyses showed that the T-DNA insertion co-segregated with the *pca22* phenotype.

Because the original *pca22* mutant was identified in the T_1_ generation, it was expected to be a gain-of-function mutant. Therefore, we examined which neighboring genes would be overexpressed owing to the four copies of the CaMV 35S enhancer elements in the activation tagging vector. In order to test the hypothesis, we used an RT–PCR approach. Through several approaches using different primer pairs in the RT–PCR analyses (Supplementary Table S1), we only detected the *At2g28625* gene, which was up-regulated in the *pca22* mutant among the three genes near to the T-DNA insertion site (Fig. 3D). These results suggested that the activated *At2g28625* gene was tightly linked with the *pca22* mutant phenotype. The *At2g28625* gene was therefore designated as *PCA22*.

**Fig. 4.**
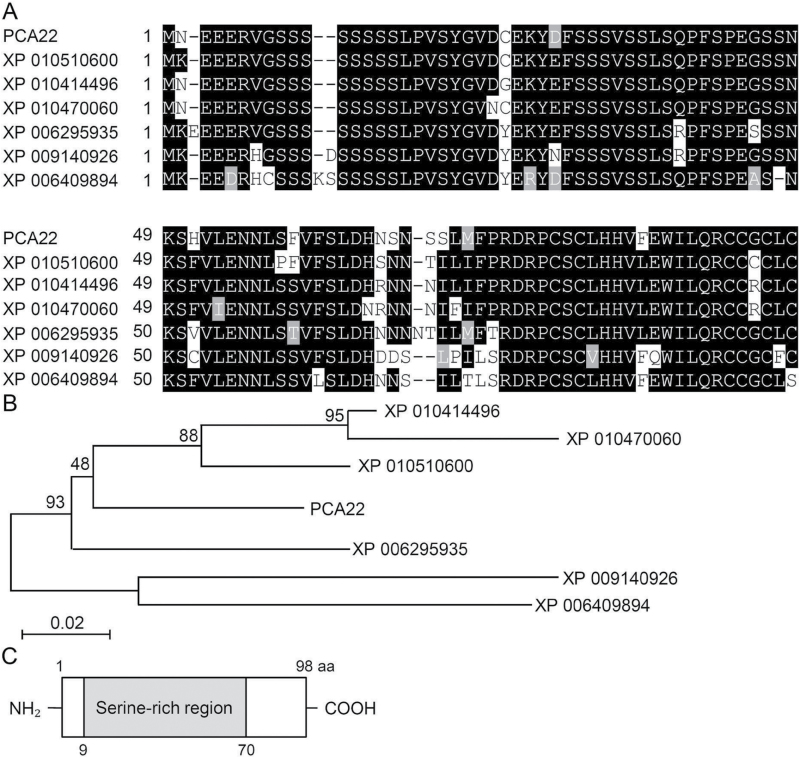
Alignment of the full-length deduced amino acid sequences of PCA22 and PCA22 orthologs from different phylogenetic origins. (A) Shown are the sequences of PCA22 (At2g28625), *Camelina sativa* XP010510600, *Camelina sativa* XP010414496, *Camelina sativa* XP010470060, *Capsella rubella* XP006295935, *Brassica rapa* XP009140926, and *Eutrema salsugineum* XP006409894. Black and gray shading indicate identical and similar amino acids, respectively. Gaps were used to optimize the alignment. (B) Phylogenetic tree depicting homology relationships among *Arabidopsis thaliana*, *C. sativa*, *C. rubella*, *B. rapa*, and *E. salsugineum* PCA22 members. Numbers at branch points indicate bootstrap values after 1000 replications. (C) The structure of the conserved regions of the PCA22 protein. The primary structure contains a serine-rich box (9–70) within its central region, which is indicated by the gray box.

To gain insight into the function of the *PCA22* gene, we attempted to isolate this gene in Arabidopsis. The isolated cDNA sequence comprised 297 bp and harbored one single ORF encoding a protein of 98 amino acids with a calculated mol. wt of 37.82 kDa, which displayed 80–89% identity with unknown proteins in *Camelina sativa*, *Capsella rubella*, *Brassica rapa*, and *Eutrema salsugineum* (Fig. 4A, B). BLAST analysis revealed the presence of only a single copy of the gene in the Arabidopsis (Col-0) genome. Based on its amino acid sequence, one highly conserved serine-rich domain in the central region was identified in the homologous proteins, although no function was assigned to this region (Fig. 4C).

### *Overexpression of* PCA22 *in the* atrzf1 *mutant*

**Fig. 5.**
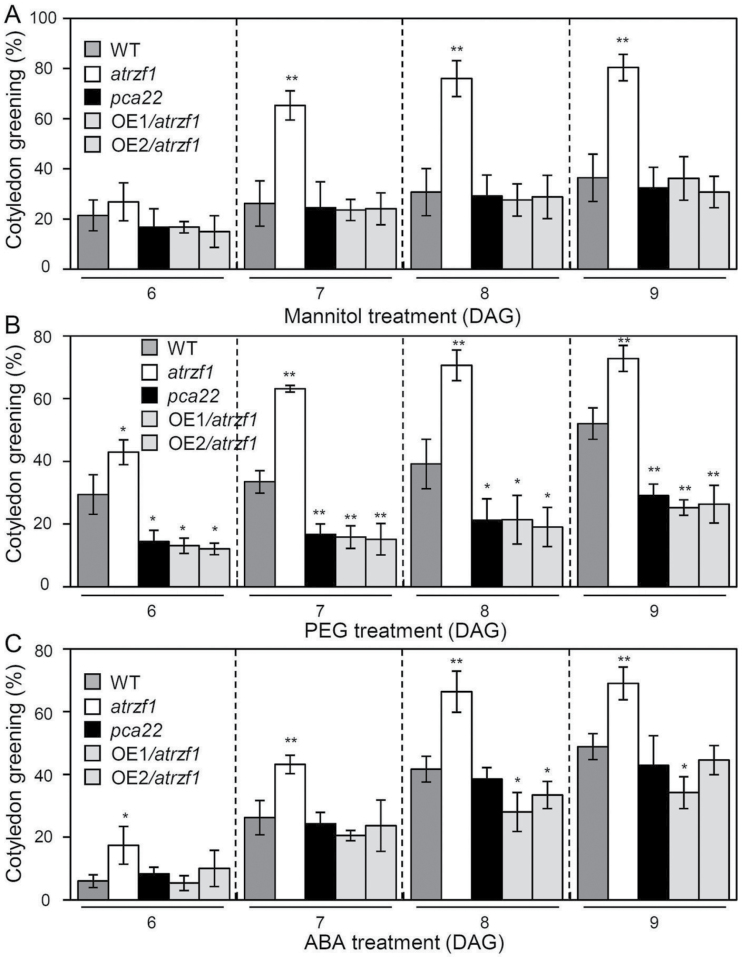
Mannitol, PEG, and ABA sensitivity as a result of *PCA22* overexpression in *atrzf1* mutants. (A–C) The effects of mannitol, PEG, and ABA treatment on cotyledon greening. Seeds were sown on MS agar plates supplemented with 400 mM mannitol (A), 10% PEG (B), or 1 μM ABA (C), and permitted to grow for 6, 7, 8, and 9 days after germination (DAG). Seedlings with green cotyledons were counted (triplicates, *n*=50 each). Error bars represent the SD. Differences between wild-type and transgenic plants grown under the same conditions are significant at 0.05>*P*>0.01 (*) or *P*<0.01 (**).

To demonstrate that activated *PCA22* is responsible for the *pca22* mutant phenotype, *PCA22* full-length cDNA under the control of the CaMV 35S promoter was transferred into the *atrzf1* mutant. Fourteen homozygous lines (T_3_ generation) were obtained, and two lines (OE1/*atrzf1* and OE2/*atrzf1*) exhibiting high levels of transgene expression (Supplementary Fig. S3A) were selected for phenotypic characterization. Comparison of the OE1/*atrzf1* and OE2/*atrzf1* transgenic plants with the *atrzf1* mutant demonstrated significantly fewer expanded and green cotyledons 6, 7, 8, and 9 days after germination (DAG) when grown on MS medium containing 400 mM mannitol, 10% PEG, or 1 μM ABA. Conversely, the cotyledon greening rate was similar between *pca22* and OE1/*atrzf1* and OE2/*atrzf1* transgenic plants (Fig. 5; Supplemetnary Fig. S3B). Taken together, our results demonstrated that overexpression of the *PCA22* gene was able to suppress the dehydration- and ABA-insensitive phenotype of the *atrzf1* mutant. We also examined the accumulation of Pro in complementation lines (OE1/*atrzf1* and OE2/*atrzf1*) under 400 mM mannitol, 10% PEG, and drought conditions. As shown in Supplementary Fig. S4A, Pro accumulation in the complementation lines was similar under dehydration conditions compared with the WT and *pca22*, but was significantly lower compared with the *atrzf1* mutant. The magnitude of Pro induction of drought conditions in *atrzf1* was greater than that of mannitol and PEG conditions, while induction of Pro in response to mannitol was similar with that of PEG treatment. The complementation results demonstrate that the overexpression of *PCA22* in the *atrzf1* line is responsible for the dehydration and ABA sensitivity of the *pca22* mutant.

Previous studies showed increases in the MDA content in plants subjected to drought stress (Wang *et al.*, 2014; You and Chan, 2015). To investigate the effect of drought stress on lipid peroxidation, we measured the MDA content in WT, *atrzf1*, *pca22*, and complementation lines (OE1/*atrzf1* and OE2/*atrzf1*). As shown in Supplementary Fig. S4B, the MDA contents did not differ substantially among the WT, *atrzf1*, *pca22*, and complementation lines under normal conditions. The MDA content in all the plants was raised by drought stress. The MDA level in *atrzf1* was lowerthan that in the WT, whereas *pca22* and complementation lines displayed significantly higher MDA contents than the WT after drought stress (Supplementary Fig. S4B). Thus, PCA22 is involved in the production of lipid peroxidation by drought stress.

### *Expression analysis of the* PCA22 *gene in Arabidopsis*

**Fig. 6.**
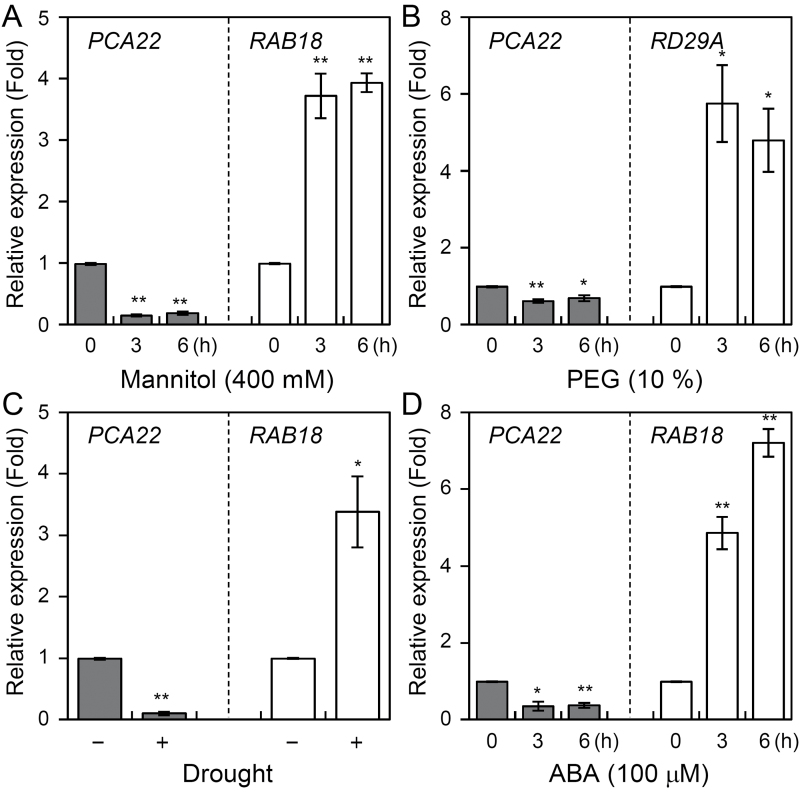
*PCA22* expression in *Arabidopsis* plants grown under abiotic stress conditions. (A–D) qPCR analyses showing the expression of *PCA22*, *RAB18*, and *RD29A* in response to abiotic stress conditions. All quantifications were made in three independent RNA samples obtained from plants treated with 400 mM mannitol (A), 10% PEG (B), drought (C), and 100 μM ABA (D) for the indicated times. Error bars indicate the SD of three independent biological samples. Differences among the expression of *PCA22*, *RAB18*, or *RD29A* in Arabidopsis seedlings with and without treatment with various abiotic stresses are significant at 0.05>*P*>0.01 (*) or *P*<0.01 (**).

To investigate the function of PCA22, we initially assessed its expression pattern. The results of qPCR experiments demonstrated that *PCA22* was expressed relatively strongly in flowers, weakly in roots, and very weakly in rosette leaves and stems (Supplementary Fig. S5). Next, in an effort to determine the *in vivo* functions of PCA22, we assessed the accumulation of *PCA22* RNA in Arabidopsis seedlings subjected to mannitol, PEG, drought, and ABA treatment using qPCR. As shown in Fig. 6, *PCA22* was reduced significantly in 10-day-old Arabidopsis seedlings within 6 h of mannitol, PEG, or ABA treatment. Samples subjected to drought stress also showed a significant reduction in *PCA22* expression in whole plants of 26-day-old seedlings (Fig. 6C). *PCA22* expression was reduced under abiotic stress, in contrast to expression of the control *RAB18* and *RD29A* genes (Sharma and Verslues, 2010), which was induced strongly (Fig. 6).

**Fig. 7.**
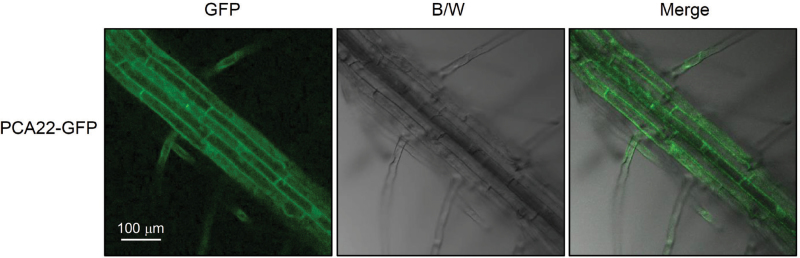
Cytoplasmic localization of PCA22. Five-day-old transgenic plants grown on half-strength MS agar medium were analyzed for GFP expression by confocal microscopy. The PCA22–GFP signal was mainly observed in the cytoplasm of the root cells. GFP, green fluorescent protein; B/W, black and white. Scale bars=100 μm. (This figure is available in colour at *JXB* online.)

To assess the subcellular localization of PCA22, a CaMV 35S promoter (35S)-driven fusion gene (35S-PCA22–GFP) from *PCA22* cDNA was constitutively expressed in Arabidopsis. Using phosphinothricin resistance segregation, we selected homozygous plants for 35S-PCA22–GFP. The fluorescence of the 35S-PCA22–GFP construct was found to be quite strong in the cytoplasm of the root cells in transgenic seedlings (Fig. 7).

### *Overexpression of* PCA22 *confers high sensitivity of Arabidopsis to ABA and dehydration stresses*

**Fig. 8.**
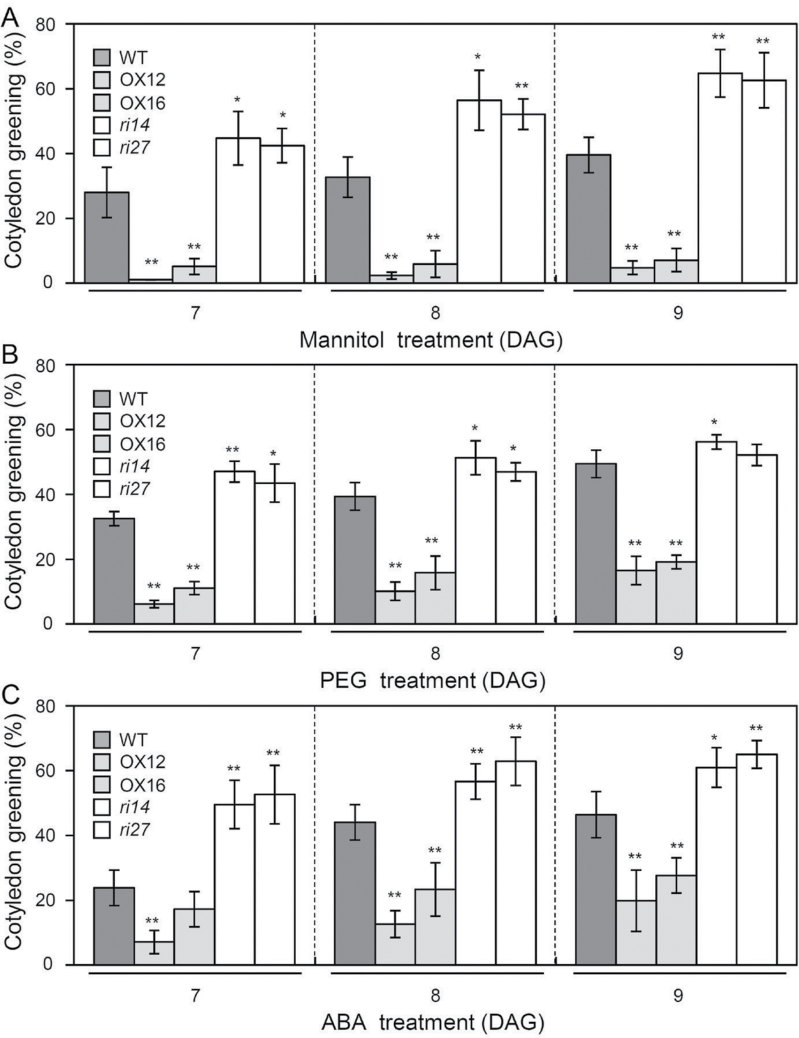
Abiotic stress sensitivity of *PCA22* transgenic plants. (A–C) Effect of mannitol, PEG, and ABA treatment on cotyledon greening. Seeds were sown on MS agar plates supplemented with 400 mM mannitol (A), 10% PEG (B), or 1 μM ABA (C), and were permitted to grow for 7, 8, and 9 days after germination (DAG). Seedlings with green cotyledons were counted (triplicates; *n*=50 each). Error bars represent the SD. Differences between WT and transgenic plants grown under the same conditions are significant at 0.05>*P*>0.01 (*) or *P*<0.01 (**).

To investigate the *in vivo* function of PCA22, *PCA22* overexpression was induced in Arabidopsis under the control of the 35S promoter. Eleven homozygous lines (T_3_ generation) were obtained, and two lines (OX12 and OX16) exhibiting high levels of transgene expression (Supplementary Fig. S6A) were selected for phenotypic characterization. In an effort to evaluate further the functional consequence of the loss of *PCA22*, RNAi lines (*pca22ri*) were generated using full-length cDNA sequences. *PCA22* expression was assessed by RT–PCR in two randomly selected independent *pca22ri* lines (*ri14* and *ri27*). *PCA22* expression was knocked down in the RNAi lines (Supplementary Fig. S6B), and the effects of mannitol, PEG, and ABA on cotyledon greening efficiency in the *PCA22* transgenic lines were tested. Seeds of WT, *pca22ri*, and *PCA22*-overexpressing plants were grown on sterile medium containing 400 mM mannitol, 10% PEG, or 1 μM ABA. The percentage of germinated seeds was calculated from the number of seedlings with cotyledon greening 7, 8, and 9 DAG. WT, *pca22ri*, and *PCA22*-overexpressing seeds germinated almost evenly under normal conditions; however, in the presence of 400 mM mannitol, ~32.7% of the WT seeds and only 4.3% (range 2.4–6.1%) of the *PCA22*-overexpressing seeds germinated for 8 DAG (Fig. 8A; Supplemetnary Fig. S6C). In contrast, ~54.3% (range 52.1–56.4%) of the *pca22ri* seeds germinated in the presence of mannitol. At 10% PEG, ~13% (range 10.1–15.8%) of *PCA22*-overexpressing seeds germinated for 8 DAG, while this increased to 39.3% and ~49.2% (range 47.1–51.3%) for WT and RNAi seeds, respectively (Fig. 8B; Supplementary Fig. S6D). When permitted to grow for 8 DAG prior to the assessment of cotyledon greening rates in the *PCA22*-overexpressing plants in response to ABA, 44% of the WT leaves expanded and turned green, as compared with ~59.8% (range 56.7–62.9%) in the *pca22ri* lines (*ri14* and *ri27*). Conversely, ~18% (range 12.7–23.3%) of two *PCA22*-overexpressing transgenic lines survived 8 DAG (Fig. 8C; Supplementary Fig. S6E). These results show that *pca22ri* lines are more likely to be insensitive to dehydration and ABA stresses than the WT. However, the *PCA22*-overexpressing plants were shown to be more sensitive to dehydration stress and ABA than the WT and *pca22ri* plants.

Pro content in different lines was also determined. Under normal, unstressed conditions, the Pro content was similar in all lines (data not shown). During drought stress conditions, a significant increase in Pro content was observed in *pca22ri* lines (*ri14* and *ri27*), but the decrease was more marked in *PCA22*-overexpressing transgenic lines (OX12 and OX16) than in the WT (Supplementnary Fig. S7). These results indicate that disruption of *PCA22* expression promotes Pro accumulation.

### pca22 *reduces pollen tube length*

**Fig. 9.**
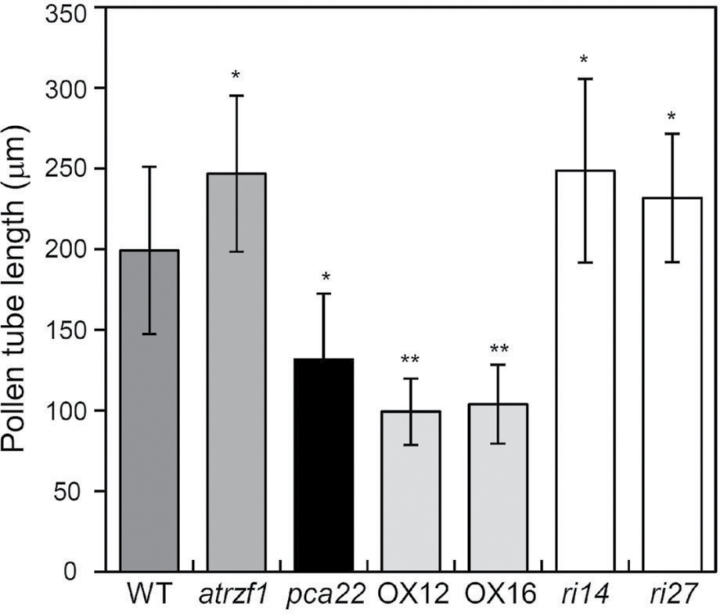
Length of *in vitro* grown WT, *atrzf1*, *pca22*, and *PCA22* transgenic pollen tubes. After 12 h pollen germination, the lengths of 150 pollen tubes from each line were measured. *pca22* and *PCA22*-overexpressing (OX12 and OX16) pollen tubes were significantly shorter than WT, *atrzf1*, and *pca22ri* (*ri14* and *ri27*) pollen tubes. Error bars represent the SD. Differences among WT, *atrzf1*, *pca22*, *PCA22*-overexpressing (OX12, OX16), and *pca22ri* (*ri14*, *ri27*) lines grown under the same conditions are significant at 0.05>*P*>0.01 (*) or *P*<0.01 (**).

Because *PCA22* transcripts accumulate at high levels, especially in flower organs (Supplementary Fig. S5), it would be interesting to evaluate whether PCA22 is associated with pollen tube growth. Several papers also reported that Pro is considered an important flowering signal during pollen tube germination and fertilization because it accumulates markedly in reproductive tissues (Schwacke *et al.*, 1999; Funck *et al.*, 2012). To determine whether PCA22 is associated with pollen tube growth, we measured pollen tubes *in vitro* after 12 h pollen germination. As shown in Fig. 9 and Supplementary Fig. S8, *pca22* pollen tubes were shorter than WT and *atrzf1* pollen tubes. However, the average length of *atrzf1* pollen tubes was longer than that of the WT pollen tubes (Fig. 9). The *atrzf1* pollen tubes were similar in appearance to *pca22ri* (*ri14* and *ri27*) pollen tubes. Quantification of the lengths of WT and *PCA22*-overexpressing (OX12 and OX16) pollen tubes revealed a significant difference in their length. Pollen from both *PCA22*-overexpressing lines formed relatively short pollen tubes (Fig. 9). From this, we concluded that PCA22 can regulate the length of pollen tubes.

## Discussion

In many plants, a strong correlation has been established between Pro accumulation and abiotic stress tolerance. Recently, the protein ubiquitination pathway has been shown to exert a strong influence on Pro accumulation, and the expression of related genes is tightly regulated depending on the environmental conditions (Chu *et al.*, 1978; Meon *et al.*, 1978; Yoshiba *et al.*, 1995; Seki *et al.*, 2002; Yang *et al.*, 2009). Therefore, modulators of this pathway may be involved in the regulation of Pro metabolism, although their individual contribution may be minimal. Previous studies have reported that AtRZF1 functions as an E3 ubiquitin ligase, which regulates Pro accumulation under drought conditions (Ju *et al.*, 2013). Using WT plants to screen for extreme drought sensitivity is difficult, and may lead to the activation of other components of the ubiquitination pathway. Therefore, we screened in the genetic background of the *atrzf1* mutant, which is insensitive to dehydration. Pro accumulation in response to drought during the vegetative stage was used as a physiological marker, which enabled the isolation of the suppressor mutants in the present study. One of the mutants isolated from this screen was characterized as *pca22* (for *proline content alterative 22*).

*pca22* mutant plants were found to possess dominant mutations, and were therefore expected to behave as gain-of-function mutants to suppress the *atrzf1* phenotype caused by induced expression of the *PCA22* gene. In fact, the Pro content detected in the *pca22* mutant was lower than that in *atrzf1* (Fig. 1). This result initially indicated that *pca22* suppressed *atrzf1*, leading to the decrease in Pro accumulation. Comparison of the *pca22* and *atrzf1* mutants revealed significant sensitivity to dehydration stress and ABA during early seedling development, whereas the cotyledon greening rate was similar in WT and *pca22* lines (Fig. 2). In addition, *pca22* plants displayed much higher sensitivity to 10% PEG during early seedling growth than did WT and *atrzf1* plants. These results showed that activation of the *PCA22* gene suppresses the *atrzf1* phenotype during seedling growth in terms of ABA and dehydration stress responses. Additionally, the complementary lines were hypersensitive to ABA and water deficit stress compared with the *atrzf1* mutant (Fig. 5). Therefore, it was believed that in complementary lines, Pro synthesis was down-regulated under drought stress. Consequently, our study also demonstrates a distinct difference in water loss, Pro content, and MDA level between *atrzf1* and *pca22* mutants (Supplementary Figs S1, S4). The leaves of the *pca22* mutant exhibited a significant increase in water loss and MDA content under drought conditions compared with *atrzf1* leaves (Supplementary Figs S1, S4). With regard to Pro content, this was higher in the *atrzf1* mutant than in the *pca22* mutant (Supplementary Fig. S4). In addition, the accumulation of Pro in the *PCA22* RNAi (*pca22ri*) lines exhibited higher levels than the WT and *PCA22*-overexpressing plants after drought treatment (Supplementary Fig. S7), which might suggest that PCA22 is responsible for the induction of leaf drought sensitivity through the modulation of lipid peroxidation and osmolytic components. In the present study, the transcript levels of dehydration-inducible genes, including *RD29B*, *RAB18*, *P5CR*, *P5CS*, and *AtMYB2*, were more markedly reduced in the *pca22* mutant than in the *atrzf1* mutant following mannitol treatment (Supplementary Fig. S2). Similarly, the transcript level of *AtbZIP11*, which is known to be down-regulated during dehydration conditions, decreased more in the *atrzf1* mutant than in WT and *pca22* mutant plants (Supplementary Fig. S2), suggesting that PCA22 can participate or amplify the signal required for regulating the expression of dehydration stress-related genes following activation of the AtRZF1-mediated signaling pathway. PCA22 was found to be a cytoplasmic protein (Fig. 7) and shared high homology with unknown proteins from *C. sativa* (Fig. 4A, B). A serine-rich region was observed at amino acid residues 9–70 in the central region of the PCA22 protein (Fig. 4C). The serine-rich domain is a common protein motif involved in defense responses, abiotic stress, growth regulation, wounding, and plant development (Sripinyowanich *et al.*, 2013; Deshmukh *et al.*, 2014). The serine-rich domain is also found in the products of the *Porteresia coarctata serine*-*rich*-*protein* (*PcSrp*) and *Capsicum annuum RING*-*finger protein 1* (*CaRFP1*) genes, whose functions are dependent on salinity and biotic stress conditions, respectively (Mahalakshmi *et al.*, 2006; Hong *et al.*, 2007). It is possible that other stress-responsive genes containing a serine-rich domain have evolved to function in the stress response.

Interestingly, in the present study, expression of *PCA22* in the dehydration-insensitive mutant *atrzf1* was found to be down-regulated when compared with that in the WT under normal conditions (Supplementary Fig. S3A). These observations indicate that decreased *AtRZF1* transcription affected downstream signal pathway transduction and conferred plant abiotic stress tolerance. Therefore, it is considered that PCA22 acts downstream of E3 ubiquitin ligase AtRZF1 in ABA and osmotic stress signaling. Conversely, a feedback loop may function through AtRZF1 and other intermediate molecules, leading to the up-regulation of *PCA22* expression. Taken together, these findings suggest that PCA22 might play a dual or distinctive role in abiotic stress responses, acting specifically as a modulator of the ubiquitination pathway or as a transducer of proline metabolism during ABA and dehydration stress signaling.

Water deficit response assays indicated that while the *pca22ri* transgenic lines were less sensitive to drought, *PCA22*-overexpressing plants were more sensitive, suggesting that PCA22 negatively regulates the drought response during early seedling development (Fig. 8).

Organ size is an important part of biodiversity and results from the interaction between organisms and their environment. As plants are sessile, the development of their organs depends greatly on the prevailing environmental conditions (Guan *et al.*, 2009). The fact that *PCA22* overexpression led to a reduction in pollen tube length (Fig. 9; Supplementary Fig. S8) suggests that PCA22 may be involved in the regulation of pollen tube length in Arabidopsis. In plants, *PCA22* was preferentially expressed in flowers, and was expressed to a weaker degree in leaves and stems (Supplementary Fig. S5). These data suggest that PCA22 may fulfill specific functions on spatial and temporal levels during plant development and/or in response to environmental stresses.

The main objective of the present study was to identify the suppressor responsible for the drought-sensitive phenotype of the *atrzf1* mutant during the early seedling stage. Analyses focused mainly on Pro accumulation under a range of applied abiotic stress conditions and resulted in the identification of *PCA22* as a suppressor gene. The ability of *PCA22* overexpression to suppress insensitivity to water deficiency in the *atrzf1* mutant clearly demonstrates a possible link between these genes and their products in the dehydration pathway. During dehydration conditions, AtRZF1 positively regulates the PCA22 molecule that exerts a negative control on Pro accumulation. Thus, loss of PCA22 leads to elevated Pro accumulation under drought conditions. Overall, further functional studies of PCA22 and AtRZF1, their target proteins, and their interactions are necessary for a complete understanding of drought response networks in plants.

## Supplementary data

Supplementary data are available at *JXB* online.

Table S1. Gene-specific primers used for TAIL-PCR, qPCR, and RT–PCR assays.

Fig. S1. Measurement of water loss in WT, *atrzf1*, and *pca22* plants.

Fig. S2. Expression of drought-regulated genes in *pca22* plants under osmotic stress.

Fig. S3. *PCA22* overexpression phenotypes in *atrzf1* mutant plants.

Fig. S4. Proline accumulation in complementation lines.

Fig. S5. Expression of the *PCA22* gene in Arabidopsis.

Fig. S6. Abiotic stress response of the *PCA22* transgenic plants.

Fig. S7. Proline content in leaves of *PCA22* transgenic lines.

Fig. S8. Overexpression of *PCA22* inhibits pollen tube growth.

## Supplementary Material

Supplementary_Figures_S1_S8_Table_S1

## Abbreviations:

AtRZF1: *Arabidopsis thaliana* Ring Zinc Finger 1
GFP: green fluorescent protein
PCA22: Proline Content Alterative 22
qPCR: quantitative real-time PCR
RT–PCR: reverse transcription–PCR
WT: wild type.
