# Supplementary material for: PCA22 acts as a suppressor of atrzf1 to mediate proline accumulation in response to abiotic stress in Arabidopsis
Source: J Exp Bot. 2017 Mar 21;68(7):1797–809. doi: 10.1093/jxb/erx069 (PMC5444443; doi:10.1093/jxb/erx069)
Supplement: Supplementary_Figures_S1_S8_Table_S1 [file erx069_suppl_Supplementary_Figures_S1_S8_Table_S1.pdf]

Figure S1

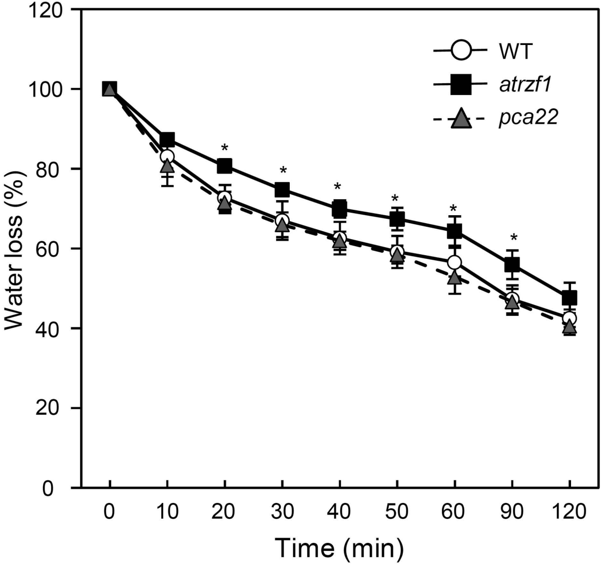

WT
atrzf1
pca22
Water loss (%)
Time (min)
0
20
40
60
80
100
120
0
10
20
30
40
50
60
90
120

Figure S2

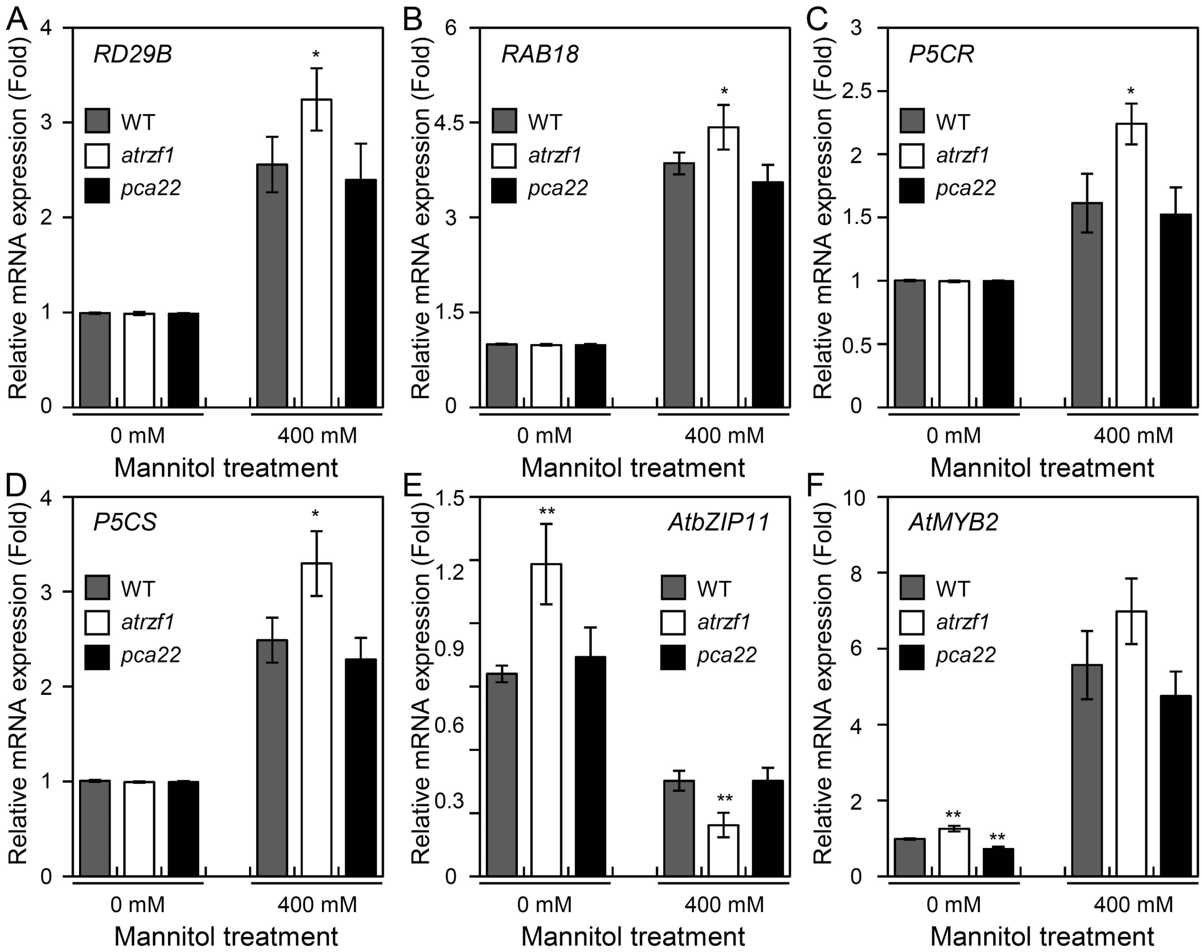
A
RD29B
B
RAB18
C
P5CR
D
P5CS
E
AtbZIP11
F
AtMYB2
WT
atrzf1
pca22
*
**
Relative mRNA expression (Fold)
0 mM
400 mM
Mannitol treatment
0
1
2
3
4
1.5
4.5
6
0.5
2.5
0.3
0.6
0.9
1.2
8
10

Figure S3

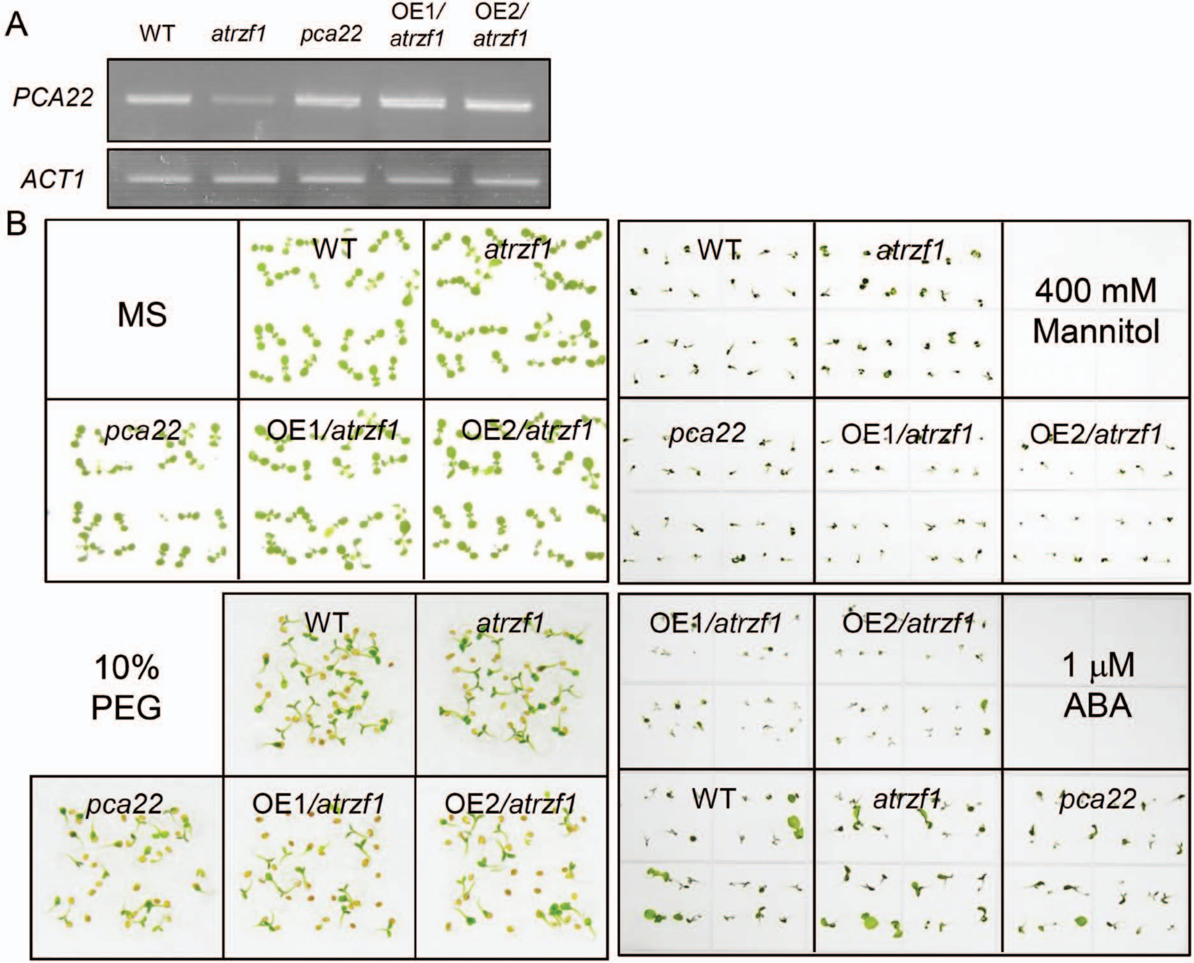

A
WT
atrzf1
pca22
OE1/ atrzf1
OE2/ atrzf1
PCA22
ACT1
B
MS
WT
atrzf1
pca22
OE1/atrzf1
OE2/atrzf1
400 mM Mannitol
WT
atrzf1
pca22
OE1/atrzf1
OE2/atrzf1
10% PEG
WT
atrzf1
pca22
OE1/atrzf1
OE2/atrzf1
1 μM ABA
OE1/atrzf1
OE2/atrzf1
WT
atrzf1
pca22

Figure S4

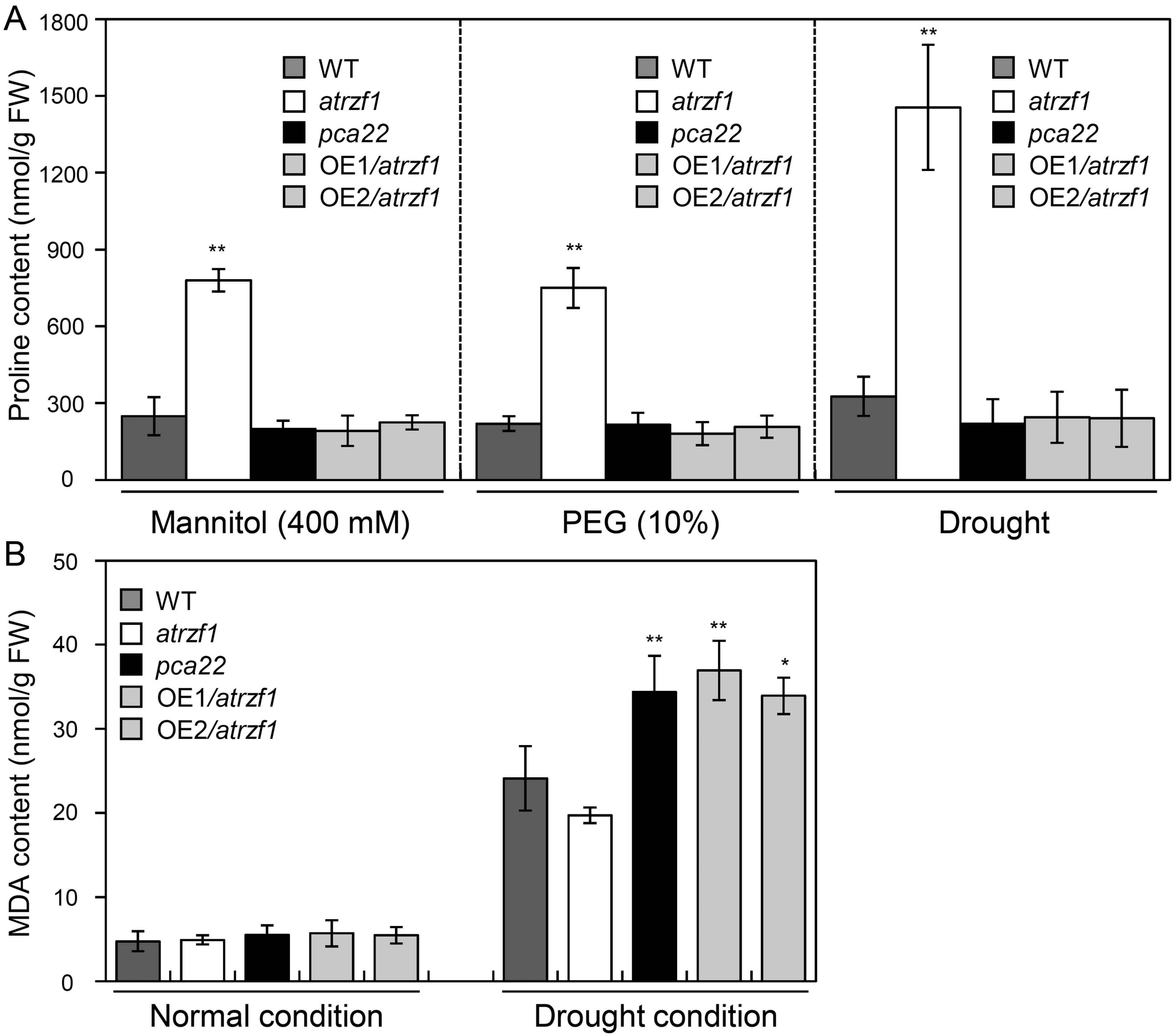
A
Proline content (nmol/g FW)
1800
1500
1200
900
600
300
0
WT
atrzf1
pca22
OE1/atrzf1
OE2/atrzf1
**
**
**
Mannitol (400 mM)
PEG (10%)
Drought
B
MDA content (nmol/g FW)
50
40
30
20
10
0
WT
atrzf1
pca22
OE1/atrzf1
OE2/atrzf1
**
**
*
Normal condition
Drought condition

Figure S5

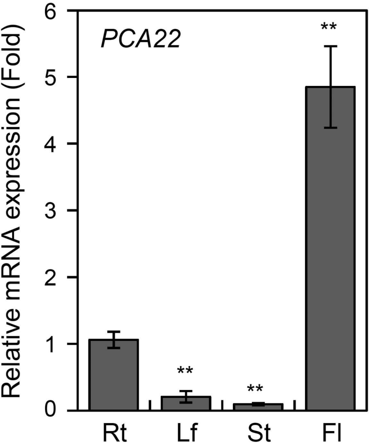

Figure S6

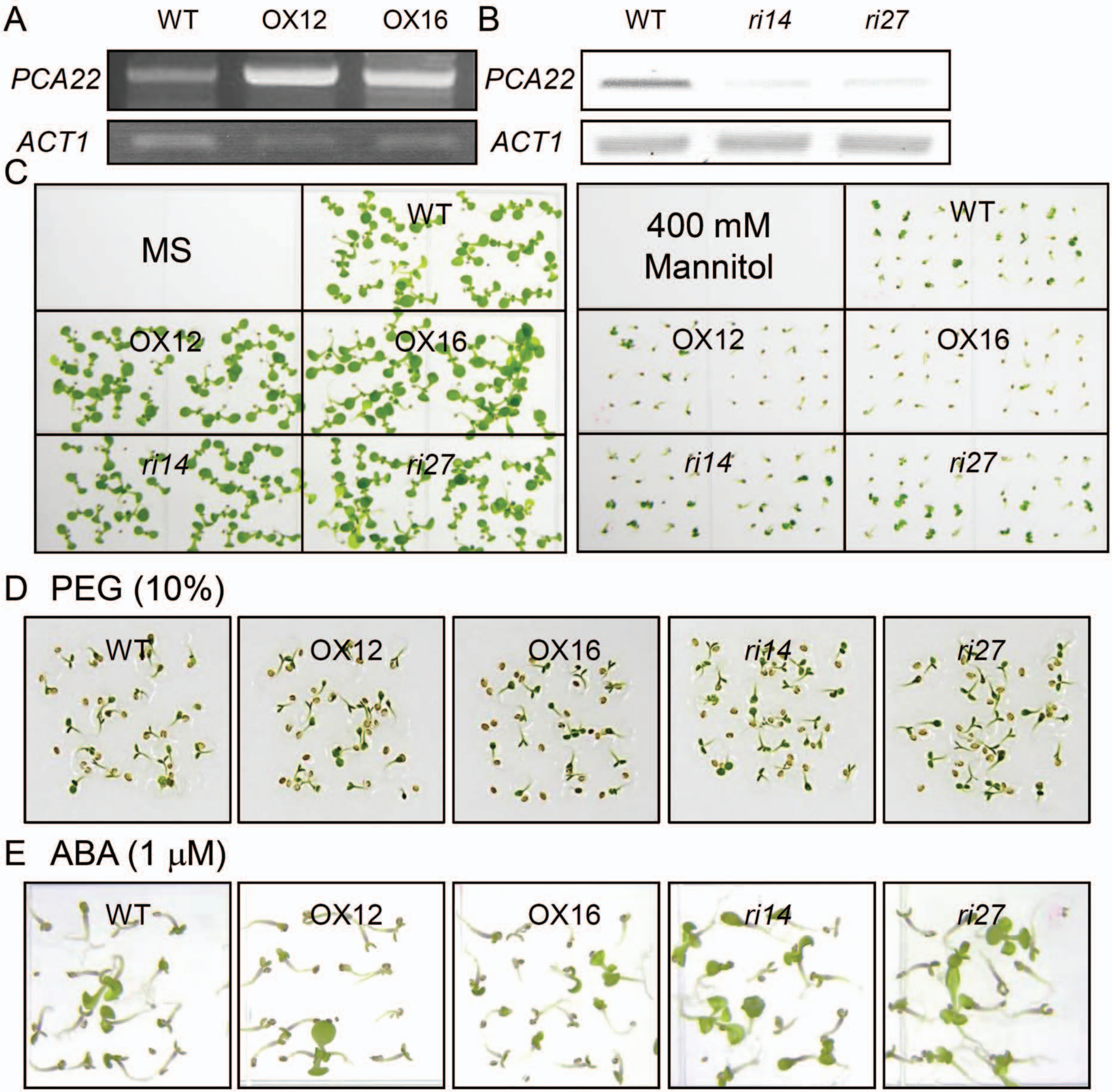
A
WT
OX12
OX16
PCA22
ACT1
B
WT
ri14
ri27
PCA22
ACT1
C
MS
WT
OX12
OX16
ri14
ri27
400 mM Mannitol
WT
OX12
OX16
ri14
ri27
D PEG (10%)
WT
OX12
OX16
ri14
ri27
E ABA (1 μM)
WT
OX12
OX16
ri14
ri27

Figure S7

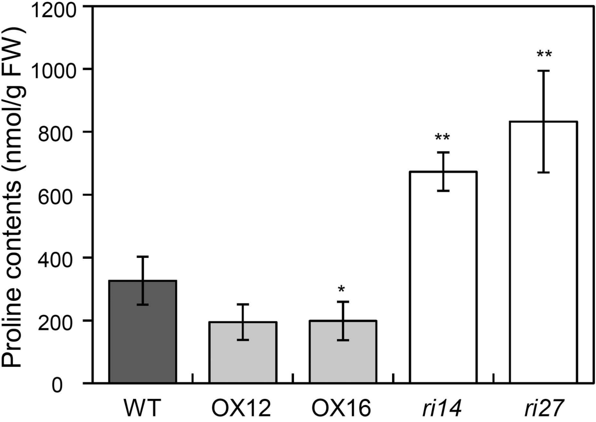

Proline contents (nmol/g FW)
1200
1000
800
600
400
200
0
*
**
**
WT
OX12
OX16
ri14
ri27

Figure S8

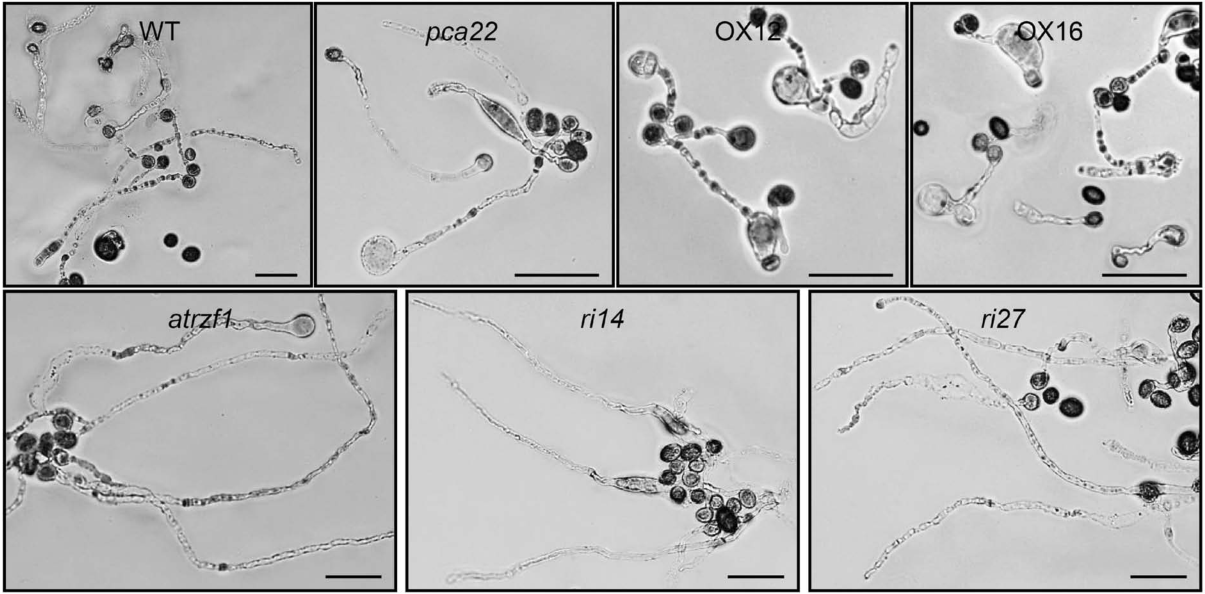

Table S1. Gene-specific primers used for TAIL-PCR, qPCR and RT-PCR assays

**Primer**	**Primer sequence (5' to 3') used for TAIL-PCR**
LB1	ATACGACGGATCGTAATTTGTC
LB2	TAATAACGCTGCGGACATCTAC
LB3	TTGACCATCATACTCATTGCTG
AD2	NGTCGASWGANAWGAA
**Gene**	**Primer sequence (5' to 3') used for qPCR**
PCA22 (At2g28625)	Forward: CTGTGAGCTACGGAGTTGACTG<br>Reverse: CTAACAAAGGCAACCACAACATC
*P5CR (*At5g14800*)*	Forward: GATGGAGATTCTTCCGATTCC<br>Reverse: CCAGCTGCAACAGAAACCAGA
P5CS1 (At2g39800)	Forward: CGACGGAGACAATGGAATTGT<br>Reverse: GATCAGAAATGTGTAGGTAGC
RAB18 (At5g66400)	Forward: CGATCCAGCAGCAGTATGAC<br>Reverse: TTCGAAGCTTAACGGCCACC
RD29A (At5g52310)	Forward: GACGGGATTTGACGGAGA<br>Reverse: CCGCCACATAATCTCTACCC
RD29B (At5g52300)	Forward: CGTCCTTATGGTCATGAGC<br>Reverse: GCCTCATGTCCGTAAGAGG
AtMYB2 (At2g47190)	Forward: ATGGAAGATTACGAGCGAAT<br>Reverse: CCACTAATCTCGGCATCCA
AtbZIP11 (At4g34590)	Forward: ATGGAATCGTCGTCGTCG<br>Reverse: CATCACACTCCAAACCAAC
At2g28620	Forward: ACCGTTTAATTCAGAGGAGA<br>Reverse: GTTCTTCATTGTAGAGCTCA
KCS12 (At2g28630)	Forward: GTGGTCGGAAAACTTCCCGA<br>Reverse: GACTTTCGGGTGGGCAATCA
AtRZF1 (At3g56580)	Forward: GAATTCATGTCAAGTATTCGGAATAC<br>Reverse: GTCGACATAGTCAAAAGGCCATCCAC
ACT1 (At1g32200)	Forward: CATCAGGAAGGACTTGTACGG<br>Reverse: GATGGACCTGACTCGTCATAC
**Primer**	**Primer sequence (5' to 3') used for T-DNA insertion site**
F1	ATACGACGGATCGTAATTTGTC
R1	GTGTGATGTGAACCAAGATTG
